# A male-biased sex ratio increases the opportunity for precopulatory sexual selection but does not change the Bateman gradient

**DOI:** 10.1093/evlett/qraf001

**Published:** 2025-02-14

**Authors:** Grant C McDonald, Danielle Edmunds, Juliano Morimoto, Stuart Wigby, Jennifer C Perry

**Affiliations:** Department of Zoology, University of Veterinary Medicine Budapest, Budapest, Hungary; Department of Biology, University of Oxford, Oxford, United Kingdom; Institute of Mathematics, University of Aberdeen, King’s College, Aberdeen, United Kingdom; Programa de Pós-graduação em Ecologia e Conservação, Universidade Federal do Paraná, Curitiba, Brazil; Department of Evolution, Ecology, and Behaviour, Institute of Infection, Veterinary & Ecological Sciences, University of Liverpool, Liverpool, United Kingdom; Department of Biology, St. Francis Xavier University, Antigonish, NS, Canada; School of Biological Sciences, University of East Anglia, Norwich, United Kingdom

**Keywords:** Bateman gradient, drosophila, opportunity for selection, polyandry, sexual network, sex ratio, sperm competition

## Abstract

Theory predicts that the sex ratio within populations should influence the strength of sexual selection, and sex ratio is often used as a proxy for sexual selection. However, recent studies challenge this relationship. We manipulated adult sex ratios in *Drosophila melanogaster* to comprehensively investigate the relationship between sex ratio and sexual selection. Consistent with theory, we found stronger sexual selection in males than females and an increased variance in male reproductive success (the opportunity for selection) in male-biased sex ratios. In addition, males faced more intense sperm competition in male-biased sex ratios, although the structure of sexual networks was largely invariant to sex ratio. Despite this, we show that sex ratios did not influence sexual selection in males as measured by the Bateman gradient. We leverage randomized null models to reconcile these results and show that the higher male reproductive variance in male-biased sex ratios may be explained by random chance in mating, rather than competitive mechanisms. Our findings indicate that caution is warranted over the long-standing assumption that sex ratio bias is a good proxy for the strength of sexual selection.

Sexual selection arises from competition among individuals of the same sex over mating and fertilization opportunities (Darwin, 1871; Parker, 1970, 2014; Shuker & Kvarnemo, 2021). The ratio of males and females in a population (i.e., sex ratio) has long been regarded as a principal feature of the social environment that influences sexual selection (Emlen & Oring, 1977; Kvarnemo & Ahnesjo, 1996). Classic sexual selection theory predicts that biases in the ratio of reproductively available males to females in a population [the operational sex ratio; (Emlen & Oring, 1977; Klug et al., 2010; Weir et al., 2011)] should accentuate sexual selection on the over-represented sex, by restricting the availability of mates and increasing the intensity of intrasexual competition over reproductive opportunities (Clutton-Brock, 2017). For example, male-biased operational sex ratios are typically expected to accentuate sexual selection on males, by increasing the ability of some males to control access to matings and fertilizations (Emlen & Oring, 1977; Kvarnemo & Ahnesjo, 1996). As a consequence, many empirical studies have used sex ratio as a proxy for the strength of sexual selection (Bath et al., 2021, 2023; Klug et al., 2010; Linklater et al., 2007; McNamara et al., 2016; Nandy et al., 2013).

However, despite evidence consistent with stronger sexual selection when sex ratios are biased (Janicke & Morrow, 2018; Kvarnemo & Ahnesjo, 1996), debate persists over the link between sex ratios and sexual selection, for several reasons. First, increased intrasexual competition at biased operational sex ratios may hamper the ability of members of the abundant sex (e.g., males) to monopolize reproductive opportunities, thereby reducing variation in polygamy and diminishing sexual selection on that sex (Klug et al., 2010; Kokko et al., 2012). Moreover, if females suffer increased sexual harassment at male-biased sex ratios, then females may be favored to reduce resistance or choosiness, which may further relax sexual selection on males (Arnqvist, 1992; Fitze & Le Galliard, 2008). Second, for practical reasons, researchers often manipulate adult sex ratios rather than operational sex ratios in experimental studies (Székely et al., 2014). However, theory based on adult sex ratios does not always predict increased sexual selection in the overrepresented sex. Instead, this theory suggests that investment in mate acquisition traits can be strongest in the rarer sex (e.g., males in female-biased populations) through enhanced mating and fertilization opportunities (Jennions & Fromhage, 2017; Kappeler et al., 2022; Liker et al., 2021; Schacht et al., 2017, 2022). As a consequence, better understanding the links between adult sex ratios and sexual selection is a key goal. Third, previous studies of the relationship between sex ratio and sexual selection have often used variance in mate acquisition (i.e., opportunities for precopulatory sexual selection) as a measure of sexual selection, rather than estimating sexual selection gradients on phenotypic traits (Jennions et al., 2012; Klug et al., 2010). This is a concern because variance-based indices vary systematically with sex ratios even when sexual selection differentials remain constant (Jennions et al., 2012; Sutherland, 1985). Fourth, sexual selection on males continues after mating via competition over fertilizations [postcopulatory sexual selection, (Parker & Birkhead, 2013). It remains unclear how sex ratios influence female polyandry and thereby shape both pre and postcopulatory episodes of selection.

Here, we directly test the influence of sex ratio on the operation of sexual selection, by evaluating the effect of sex ratio on the opportunity for pre and postcopulatory sexual selection and the relationship between mating and reproductive success (i.e., Bateman gradients, Bateman, 1948; Jones, 2009) in male and female *Drosophila melanogaster*. Male *D. melanogaster* that mate with more females have higher reproductive success (Davies et al., 2023; Morimoto et al., 2019; Pischedda & Rice, 2012) and females exhibit polyandry (Imhof et al., 1998), allowing sexual selection to operate before mating and after mating via sperm competition and cryptic female choice (Gilchrist & Partridge, 2000; Morimoto et al., 2019; Snook & Hosken, 2004). We experimentally constructed groups at a male-biased, equal, or female-biased adult sex ratio. If the sex ratio is central to determining the ability of males to monopolize mating and, ultimately, fertilizations, then we predict that as the sex ratio becomes biased towards males, variance-based indices of mating and reproduction (i.e., opportunities for selection) should increase in males. Bateman gradients and postcopulatory sexual selection might also vary with sex ratio if sex ratios influence rates of female polyandry (e.g., via increased male harassment of females) and patterns of sperm competition in sexual networks. If, instead, male-biased sex ratios increase female polyandry and thereby reduce male paternity share, then Bateman gradients may decrease in male-biased groups. In females, we predicted that sexual selection would be considerably weaker compared with males and largely unresponsive to sex ratio because in this species, females are not usually limited by access to mates.

## Material and methods

### Experimental animals

Flies were maintained at 25 ^o^C and uncontrolled humidity on a 12:12 hr light:dark cycle. We used a Dahomey wild-type population and two mutant lines: one homozygous for the recessive *w*^*1118*^ allele, a loss-of-function allele for the X-linked *white* gene that confers white eyes in homozygotes (hereon “*white”*), and another homozygous for the recessive *sparkling*^*poliert*^ allele, which produces a rough-looking eye phenotype [hereon “*spa”*; (Fu et al., 1998)]. Stock populations for mutant lines were created by serially back-crossing *w*^*1118*^ or *sparkling*^*poliert*^ alleles into the Dahomey background for five generations (Perry et al., 2016) and were maintained on standard food media in large population cages with overlapping generations.

To generate experimental flies, we transferred *white* and *spa* eggs from stock populations to separate vials or bottles (50/vial or 200/bottle) containing standard food media. We collected *white* males and females and *spa* males on ice as virgins and housed them in same-sex vials of 10–20 individuals. To differentiate individuals in behavioral trials we marked individuals with a small dot of acrylic paint on the dorsal thorax at 1–4 days post-eclosion under CO_2_ anesthesia. *spa* males were marked with a black dot and designated as ‘focal’ males. *white* rival males and females were randomly assigned a red, yellow, white, green, or orange dot. We designated yellow-painted females as “focal” females. Paint color did not influence the number of copulations (rival males: $\chi_{4}^{2}$ = 2.6, *p* = 0.618; females: $\chi_{4}^{2}$ = 1.3, *p* = 0.858). Males and females were 5–8 days old when experiments began.

**Figure 1.**
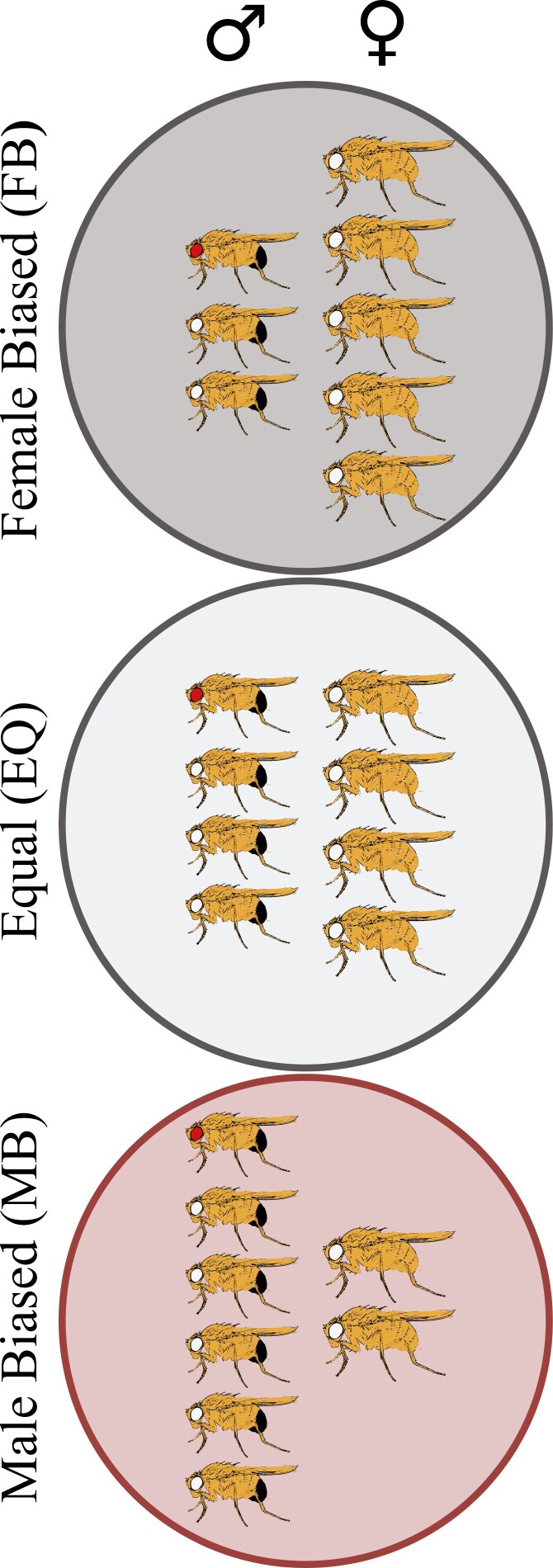
Experimental sex ratio manipulations in *Drosophila melanogaster*. Diagram representing adult sex ratio treatments. Each vial contained one red-eyed focal male and multiple rival males.

We assembled groups of 8 adult flies at either a male-biased (6 males:2 females), equal (4 males:4 females) or female-biased (3 males:5 females) adult sex ratio (Figure 1). In total, we studied 82 groups (Male-biased *n* = 28, Equal *n* = 27, Female-biased *n* = 27) after excluding vials in which individuals died or escaped. Each group had one focal red-eyed *spa* male which competed with white-eyed rival *white* males (Figure 1). We performed the experiment in three blocks in which each block contained a similar number of vials from each sex ratio treatment. Groups were enclosed in vials containing food media with live yeast grains. Our female-biased treatment was not symmetrical to our male-biased treatment to minimize the possibility that unrepresentative data points might arise in a system with only two males (e.g., if a poor-condition rival male was unable to provide sufficient mating competition to the focal male).

Focal *spa* males sire red-eyed daughters, while *white* males sire white-eyed daughters, which allowed us to differentiate daughters sired by focal and rival males (Morimoto et al., 2019). A similar proportion of daughters to sons was produced (50.27% [49.62–50.91 95% CI], binomial test, *p* = 0.420) across sex ratios ($\chi_{2}^{2}$ = 3.5, *p* = 0.177). Eye mutations can impair visual acuity. For this reason, we used *spa* mutants as focal males, so that both focal and rival males would have impaired vision (rather than impaired vision in white-eyed rival males only). The number of focal *spa* male copulations did not differ from that expected if focal and rival males had equal mating ability in male-biased and female-biased groups, and focal males instead had a lower number of copulations than expected in equal sex ratios (Supplementary Figure S1a). We also examined whether the number of offspring produced by focal males differed from expectations of equal offspring production by rivals and focals. The results suggested that focal males were disadvantaged in reproduction in equal and female-biased sex ratios, but counterintuitively focal and rival males produced a similar number of offspring in the potentially more competitive male-biased sex ratios (Supplementary Figure S1b).

### Behavioral and reproductive assays

We observed groups of flies for 4 hr per day for three consecutive days. We set up groups at 0 hr Zeitgeber time on day 1 by aspirating groups of females and males into new food vials containing yeast. A single observer recorded all matings and the identity of copulating flies. On each day, we separated males and females after observation using light CO_2_ anesthesia, housing males from each group together and females individually in yeasted egg-laying vials, allowing us to measure the reproductive success of individual females and the reproductive success and paternity share of each focal male from his number of daughters. On days 2 and 3, groups were re-assembled using the same individuals. Although male seminal fluid proteins can render females unreceptive to mating for up to 5 days post-mating (Hopkins & Perry, 2022), females will sometimes remate within this period (Imhof et al., 1998). Therefore, we expect our adult sex ratio treatments to reflect operational sex ratios throughout our experiment, with a shift towards more male-biased (or less female-biased) operational sex ratios in all treatments after females mate for the first time and become refractory. We incubated egg-laying vials at 25 ^o^C for 12–14 days to allow offspring to eclose before freezing and counted offspring using the eye color of daughters to assess focal male paternity. Unless stated otherwise, we analyze results across all 3 days combined.

### Measures of male and female reproductive success

To assess the influence of sex ratio on sexual selection, we measured male total reproductive success (*T*), calculated as the total number of daughters produced by each focal male. In addition, we measured the individual components of male reproductive success: *T = M × N × P*, where *M* is the number of mating partners per male, *N* is the average fecundity of mating partners and *P* is the proportion of his partners’ eggs that he fertilizes (i.e., paternity share) (Collet et al., 2012; Marie-Orleach et al., 2024; McDonald et al., 2013; Turnell & Shaw, 2015; Webster et al., 1995). Together, sexual selection operating on variation in the number of mating partners (*M)* and partner fecundity (*N*) represents precopulatory sexual selection on mating with more or fewer mates, and with more or less fecund partners, respectively. Variation in paternity share (*P*) arises when females obtain sperm from multiple males (i.e., polyandry) and sexual selection on paternity share represents postcopulatory sexual selection (Collet et al., 2012; Dougherty et al., 2016).

We were able to measure the number of mating partners for all males (rivals and focals), whereas reproductive success, partner fecundity, and paternity share were restricted to focal males. Because focal male reproductive success was estimated as his number of daughters, we also evaluate partner fecundity and paternity share in terms of the average number of daughters produced by a male’s female partners and the proportion of those daughters sired by the focal male.

For females, we estimated total reproductive success as the total number of offspring (sons and daughters) produced per female. We measured the female number of mating partners as the number of mating partners per female.

We characterized the influence of sex ratio on overall reproduction and mating per vial, including the total number of copulations and the total number of offspring (Supporting Information).

### Analyses

#### The effect of sex ratio on the opportunity for sexual selection

We assessed the influence of sex ratio on the potential strength of sexual selection using standardized variances in reproductive measures for focal males and females.

For focal males, we calculated the opportunity for selection (*I*_*T*_) as the standardized variance in in male reproductive success (Table 1). Because reproductive success can depend on a male’s number of mates, the average fecundity of those mates, and his paternity share, we also assessed the influence of sex ratio on the opportunity for precopulatory sexual selection on mate number (*I*_*M*_, the standardized variance in mating success, Table 1) and on partner fecundity (*I*_*N*_, the standardized variance in average mate fecundity, Table 1), and the opportunity for postcopulatory sexual selection (*I*_*P*_, the standardized variance in paternity share, Table 1). Calculations of *I*_*N*_ and *I*_*P*_ included only focal males that mated.

**Table 1.** Description of sexual selection metrics.

**Variable**	**Description**	**Formula**
Opportunity for selection (*I*_*T*_)	Standardized variance in reproductive success (*T*, i.e., total number of offspring produced by individuals). Represents the upper limit to the potential strength of selection operating through variance in reproductive success.	$VAR_{T}/{\bar{T}}^{2}$
Opportunity for precopulatory selection on mate number (*I*_*M*_)	Standardized variance in the number of mating partners per individual (*M*, i.e., the total number of different mating partners per individual).	$VAR_{M}/{\bar{M}}^{2}$
Opportunity for precopulatory selection on partner fecundity (*I*_*N*_)	Standardized variance in the average fecundity of a male’s female mating partners (*N*, i.e., the average number of offspring produced by a male’s female partners).	$VAR_{N}/{\bar{N}}^{2}$
Opportunity for postcopulatory selection (*I*_*P*_)	Standardized variance in male paternity share (*P*, i.e., the proportion of a male’s partners offspring that he fertilized).	$VAR_{P}/{\bar{P}}^{2}$
Bateman Gradient (*β*_M_)	Slope of the regression of mean-standardized reproductive success (*T*) on mean standardized number of mating partners (*M*).	$T∼\beta_{M}\times M+covariates$
Jones Index	Represents the maximum potential sexual selection gradient on a precopulatory trait.	$\beta_{M}\sqrt{I_{M}}$
Sperm Competition Intensity Index (SCI)	Calculated as the harmonic mean polyandry of a male’s female partners. A value of 1 indicates a male has complete exclusivity with his female partners and higher values indicate that a male’s female partners are on average more polyandrous (i.e., mate with more males).	$1/\frac{1}{M}\left(\overset{M}{\underset{i}{\sum }}\frac{1}{k_{i}}\right)$ , where *M* is a male’s number of female mating partners, and *k*_*i*_ is the number of mating partners for that male’s *i*_*th*_ female partner
Sperm Competition Intensity Correlation (SCIC)	Slope of the regression of mean-standardized male sperm competition intensity (SCI) on mean standardized number of mating partners (*M*). Positive values indicate males with more mating partners also on average mate with the most polyandrous females. Negative values indicate males with more mating partners on average mate with the least polyandrous females.	SCI∼SCIC×M+covariates

For focal females, we assessed the impact of sex ratio on the opportunity for selection (*I*_*T*_) and the opportunity for precopulatory sexual selection on mate number (*I*_*M*_) (Table 1). We focused on focal females so that our analysis was comparable for males and females.

We tested for significant differences in opportunities among sex ratios using 95% confidence intervals from 1,000 bootstrapped iterations of the data (Davison & Hinkley, 1997) and considered differences between sex ratios significant when there was no overlap of 95% confidence intervals (Morimoto et al., 2019).

Theory indicates that differences in *I*_*M*_ between sex ratios can be driven by chance variation in mating alone (Jennions et al., 2012; Klug & Stone, 2021). For example, male-biased groups may show a significantly higher male *I*_*M*_ compared with female-biased groups, even when mating is random (i.e., when the identity of individuals that mate is determined by chance rather than by their phenotype; Jennions et al., 2012). We therefore generated null expectations for *I*_*M*_ for males and females assuming random patterns of mating. Randomizations used the observed total number of copulations for each vial, but randomly allocated copulations across all males and females and recalculated *I*_*M*_ for each vial, before calculating mean *I*_*M*_ across vials. We repeated this process 10,000 times for each sex ratio and compared randomized null distributions of mean vial *I*_*M*_ with the mean *I*_*M*_ observed, following Ruxton & Neuhäuser, (2013).

#### The effect of sex ratio on the Bateman gradient

We next assessed how pre and postcopulatory processes translate into reproductive success for males and females.

For focal males, we estimated the strength of selection on mate acquisition as the mean-standardized gradient of reproductive success on mate number [Bateman gradients, (Anthes et al., 2016; Bateman, 1948; Jones, 2009), Table 1]. We used a linear model with Gaussian error distribution with mean-standardized focal male reproductive success as a response variable. Explanatory variables included sex ratio and experimental block as categorical variables, mean-standardized number of mates and the interaction between sex ratio and number of mates to test for differences in the slope of the Bateman gradient among treatments. We included mean-standardized total vial productivity (total offspring per vial) to account for variation in female fecundity among vials.

We then compared among sex ratio treatments the maximum potential sexual selection gradient on a precopulatory male trait (i.e., the Jones index, Table 1), which takes into account both the variance in number of mates and its relationship with reproductive success (Henshaw et al., 2016; Jones, 2009) (Table 1). We estimated the Jones index as the variance-standardized gradient of reproductive success on number of mates (Henshaw et al., 2018; McDonald & Pizzari, 2018; Morimoto et al., 2019) using a linear model with Gaussian error distribution. Mean-standardized focal male reproductive success was the response variable. Explanatory variables included sex ratio and experimental block, variance-standardized number of mates, and the interaction between sex ratio and number of mates to test for differences in the Jones index between treatments. We again included mean-standardized total vial productivity.

We also calculated variance-standardized multivariate selection gradients that measure the effect of one component (e.g., number of mates) on reproductive success while controlling for the effect of the other components (e.g., partner fecundity and paternity share). This approach allows us to examine how each component of male reproductive success contributes to his overall offspring number and whether different components are more important in some sex ratios. We used a linear model with Gaussian error distribution with mean-standardized focal male reproductive success as a response variable. This model included sex ratio, variance-standardized number of mates, partner fecundity and paternity share, the interaction between sex ratio and number of mates, partner fecundity and paternity share to test for differences in slopes among sex ratio treatments, and experimental block. Due to a strong correlation between vial productivity and the fecundity of female partners, for multivariate selection gradients we included residual total vial productivity calculated from the regression of partner fecundity on total vial productivity.

For females we also calculated the mean-standardized Bateman gradients and the Jones index. Models for females followed the same structure as for males.

#### Sperm competition intensity and sexual networks

To assess how sex ratios influence sperm competition, we first calculated the sperm competition index for all mated males from sexual networks within vials (Figure 4a, Table 1, Supplementary Figure S2). Male sperm competition index is calculated as the harmonic mean polyandry of a male’s female partners (Table 1) (McDonald & Pizzari, 2016; Shuster & Wade, 2003). A higher sperm competition index indicates that a male’s female partners are on average more polyandrous (i.e., mate with more males, Table 1). To compare the sperm competition index between sex ratios we used a Gaussian mixed-effects linear model (LMM) with square root transformed sperm competition index as a response variable. Models included sex ratio and experimental block and a random effect for vial identity to account for nonindependence of males within vials.

**Figure 2.**
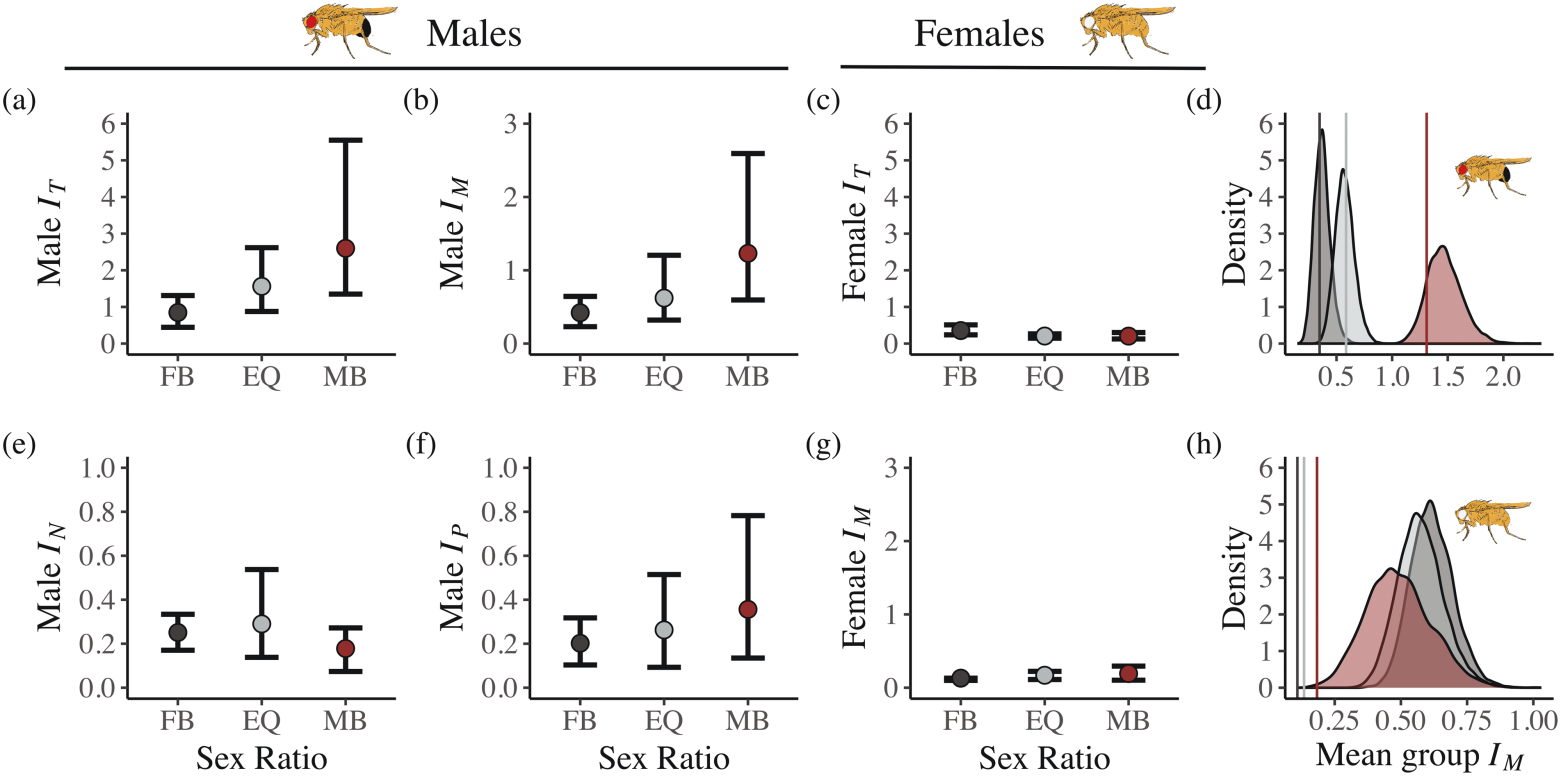
The influence of sex ratio on the opportunity for selection. For focal males, panels show (A) the opportunity for selection (*I*_*T*_), (B) the opportunity for precopulatory sexual selection on mate number (*I*_*M*_), (E) the opportunity for precopulatory sexual selection on partner fecundity (*I*_*N*_) and (F) the opportunity for male postcopulatory sexual selection (*I*_*P*_). For focal females, panels show (C) the opportunity for selection (*I*_*T*_) and (G) the opportunity for precopulatory sexual selection on mate number (*I*_*M*_). FB = female-biased groups, EQ = equal sex ratio groups, MB = male-biased groups. Error bars show bootstrapped 95% confidence intervals. (D and H) Density plots for males (d) and females (h) show the null distribution of mean group (vial) *I*_*M*_ values based on randomizations assuming random mating between all males and females within vials. Vertical lines show the observed mean *I*_*M*_ across vials for each sex ratio.

**Figure 3.**
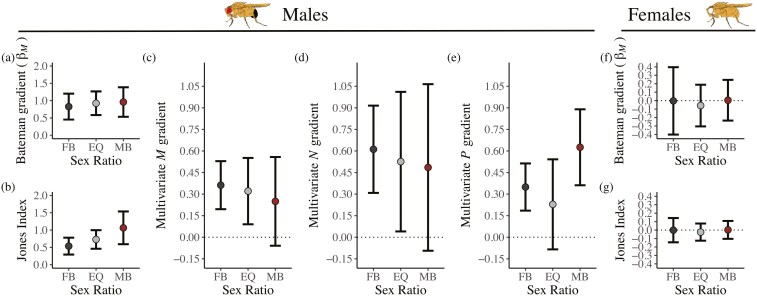
The influence of sex ratio on Bateman gradients. For focal males, panels show (A) the slopes of mean-standardized Bateman gradients ($\beta_{M}$), (B) the Jones index and variance-standardized multivariate gradients of reproductive success on (C) number of mates (*M*), (D) partner fecundity (*N*) and (E) paternity share (*P*). For focal females, panels show (F) the slopes of mean-standardized Bateman gradients ($\beta_{M}$) and (g) the Jones index. FB = female-biased groups, EQ = equal sex ratio groups, MB = male-biased groups. Error bars show 95% confidence intervals.

**Figure 4.**
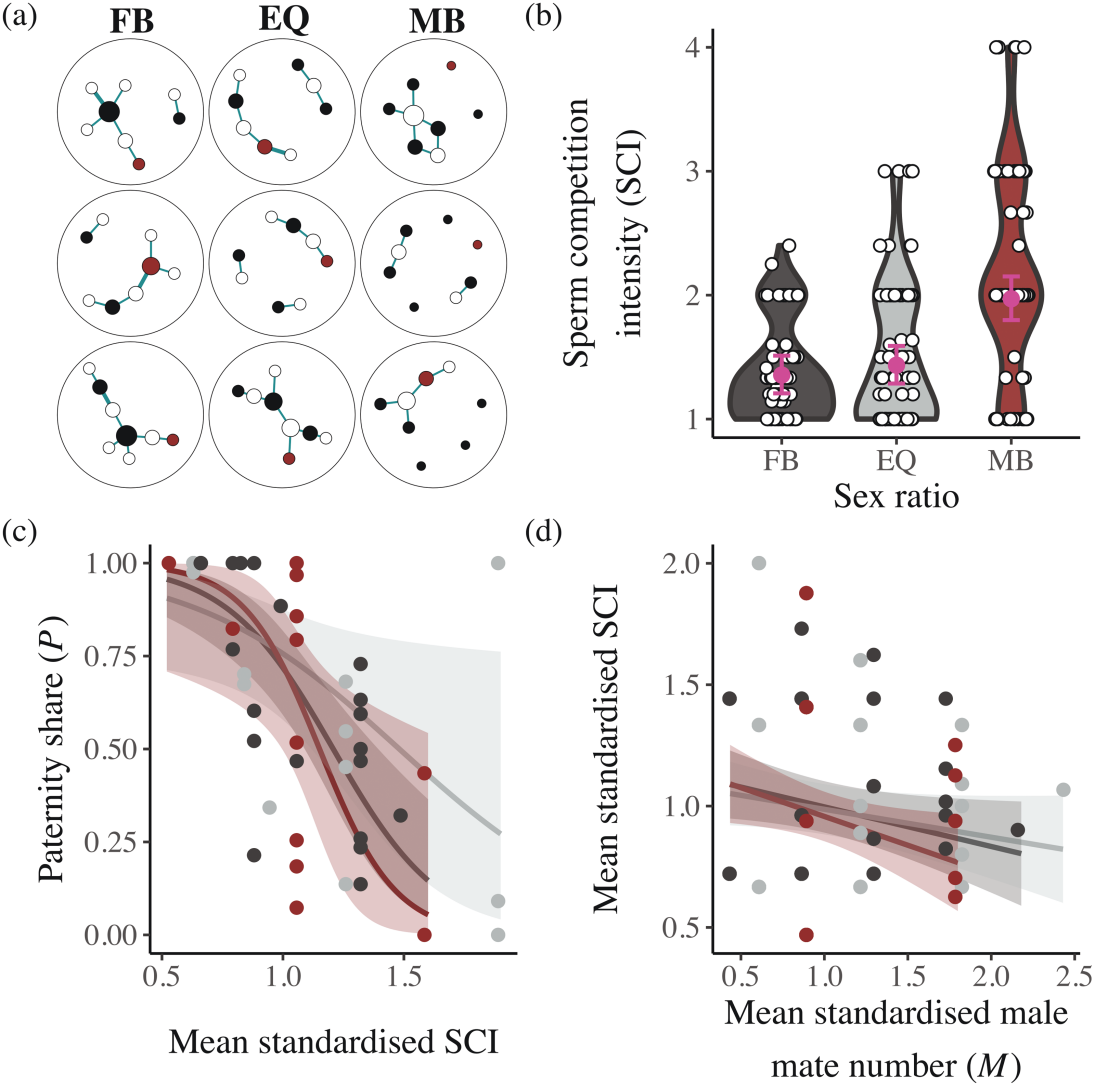
The influence of sex ratio on sperm competition. (A) A representative sample of nine sexual networks from female-biased (FB), equal (EQ), and male-biased (MB) sex ratio groups. Red nodes are focal males, black nodes are rival males and white nodes are females. Links between nodes indicate pairs mated and the thickness of links reflects the number of copulations between pairs. Node size is scaled to the number of mates per individual. (B) Violin plots show the sperm competition intensity index (SCI) of all males (focal and rival). White points show values for individual males. Pink points and bars show means and 95% confidence from mixed-effects models. (C) The relationship between focal male SCI and focal male paternity share (*P*). Each point is a focal male. (D) The relationship between male number of mates (*M*) and SCI (i.e., the Sperm Competition Intensity Correlation, SCIC) for all males (focal and rival). Lines and shaded areas represent predictions and 95% confidence intervals from models.

Second, we assessed the relationship between the number of mates per male and his sperm competition index (termed the sperm competition correlation, SCIC, Table 1) and whether this relationship varied between sex ratios. Negative SCIC values would suggest that the males most successful in mating also compete with fewer males in postmating competition, which is expected to result in reinforcing pre and postcopulatory sexual selection (McDonald, 2023). We used a Gaussian LMM of mean-standardized sperm competition index as a response variable. Explanatory variables included mean-standardized number of mates, sex ratio and the interaction between number of mates and sex ratio to test whether the slope of the sperm competition index on number of mates (i.e., SCIC) varied among sex ratios. The model also included experimental block as a fixed effect and vial identity as a random effect.

Finally, we assessed whether male sperm competition intensities predicted focal male reproductive success and paternity share and whether these patterns varied among sex ratios. Models for reproductive success used a Gaussian error distribution and included male number of mates, sperm competition index, and sex ratio as a categorical variable, as well as the interaction between sex ratio with number of mates and the sperm competition index. Models for paternity share used a quasibinomial error structure and included sperm competition index, sex ratio and the interaction between sex ratio and the sperm competition index. Both models also included total vial productivity and experimental block.

All standardizations were conducted across vials within each sex ratio. All analyses were conducted in R (R Core Team, 2022). Mixed models were constructed using package “lme4” version 1.1-30 (Bates et al., 2015). We used post-hoc Tukey tests for pairwise comparisons between sex ratios using package “emmeans” version 1.8.0 (Lenth, 2022).

## Results

### The effect of sex ratio on the opportunity for selection

As expected sex ratio strongly influenced group mating activity and patterns of reproductive success. The overall number of copulations was highest under a female-biased sex ratio and lowest under a male-biased sex ratio (Supplementary Material, Figure S3). Similarly, the number of mating partners per male was higher under a female-biased sex ratio, whereas the number of mating partners per female was higher under a male-biased sex ratio (Supplementary Material, Figure S3). As expected, the total number of offspring per vial was lower under a male-biased sex ratio, but the average reproductive success per female did not vary among sex ratios (Supplementary Material, Figure S4).

Our results support the prediction that more male-biased sex ratios increase the opportunity for selection in males (*I*_*T*_, Table 1). We found a significantly greater focal male *I*_*T*_ in male-biased relative to female-biased sex ratios (*I*_*T*_ [95% range of bootstrap]: male-biased = 2.600 [1.351, 5.550], equal = 1.562 [0.876, 2.617], female-biased = 0.849 [0.444, 1.311], Figure 2a).

In addition, for focal males we found a trend for a greater opportunity for precopulatory selection on mate number (*I*_*M*_, Table 1) in male-biased sex ratios compared to equal and female-biased sex ratios, although confidence intervals overlapped (Figure 2b). However, null models indicated that the higher male *I*_*M*_ in male-biased sex ratios is expected by random patterns of mating alone (Figure 2d). Furthermore, in no sex ratio was the observed *I*_*M*_ larger than null expectations (Figure 2d, 10,000 randomizations, male-biased *p*_*rand*_ = 0.278, equal *p*_*rand*_ = 0.852, female-biased *p*_*rand*_ = 0.773), suggesting that changes in average male mating frequencies alone likely drive trends in male *I_M_*, rather than any increase in male mate monopolization. Qualitative differences in *I*_*M*_ among sex ratios were broadly consistent across days (Supplementary Information, Figure S5).

Similarly, the opportunity for postcopulatory selection in focal males (*I*_*P*_; Table 1) was larger in a male-biased than female-biased sex ratio, although confidence intervals overlapped (Figure 2f, Supplementary Figure S6). Our results also revealed no influence of sex ratio on the opportunity for precopulatory selection on focal male ability to mate with more fecund partners (*I*_*N*_, Table 1, Figure 2e, Supplementary Figure S7).

As predicted, the opportunity for selection on focal females (*I*_*T*_) was lower relative to males in all sex ratios and was not influenced by sex ratio (Figure 2c). The opportunity for precopulatory selection on mate number (*I*_*M*_) was likewise substantially lower for focal females relative to males and was not influenced by sex ratio (Figure 2g). Consistent with results for males, null models for female *I*_*M*_ predicted higher female *I*_*M*_ in female-biased sex ratios and lower female *I*_*M*_ in male-biased sex ratios (Figure 2h). However, in contrast to males, female *I*_*M*_ was consistently lower than null expectations in all sex ratios, suggesting that variance in the number of mates females obtain is typically lower than that expected from random mating alone (Figure 2h; 10,000 randomizations, male-biased *p*_*rand*_ = 0.004, equal *p*_*rand*_ < 0.001, female-biased *p*_*rand*_ < 0.001).

### The effect of sex ratio on the Bateman gradient

We detected positive selection on the number of mates that focal males obtained: mean-standardized Bateman gradients (Table 1) were positive (*F*_1,75_ = 70.996, *p* < 0.001, Supplementary Table S1). However, contrary to predictions, the strength of positive selection on a male’s number of mates was not influenced by sex ratio (*F*_1,73_ = 0.2, *p* = 0.801; Figure 3a, Supplementary Figure S8, Table S1). This indicates that the fitness benefit males gained from acquiring more mates did not differ among sex ratios. In contrast, we found a nonsignificant tendency for a higher Jones index (Table 1) in male-biased groups (*F*_2,73_ = 2.8, *p* = 0.068; Figure 3b, Supplementary Table S2), indicating that the maximum potential sexual selection gradient on precopulatory male traits may increase under a male-biased sex ratio, in line with the prediction that male-biased sex ratios accentuate selection on males.

Multivariate gradients indicated that all components of focal male reproductive success (i.e., number of mates, partner fecundity, and paternity share) significantly and positively predicted focal male reproductive success (number of mates: *F*_2,47_ = 45.9, *p* < 0.001; partner fecundity: *F*_2,47_ = 33.3, *p* < 0.001; paternity share: *F*_2,47_ = 53.2, *p* < 0.001, Supplementary Table S3). Multivariate gradients for number of mates and partner fecundity were not influenced by sex ratio (number of mates × sex ratio: *F*_2,42_ = 0.1, *p* = 0.909, partner fecundity × sex ratio: *F*_2,42_ = 0.3, *p* = 0.773, Figure 3c-d, Supplementary Table S3), indicating no differences in the contribution of precopulatory sexual selection to a male’s offspring production among sex ratios. In contrast, the interaction between paternity share and sex ratio reached statistical significance after excluding males that mated only with sterile females (*F*_2,41_ = 3.1, *p* = 0.054; excluding sterile: *F*_2,40_ = 3.7, *p* = 0.033). This pattern appeared to be driven by a steeper paternity share gradient in male-biased groups (Figure 3e). This result is consistent with an increase in the importance of postcopulatory sexual selection in male-biased groups.

In contrast to males, focal female Bateman gradients provided no evidence that mating with more males increased reproductive success (*F*_1,75_ = 0.1, *p* = 0.816; Figure 3f, Supplementary Table S4). This pattern was consistent across sex ratios (*F*_1,73_ = 0.0, *p* = 0.953; Figure 3f, Supplementary Table S4). Similarly, the female Jones index was not significantly positive (*F*_1,73_ = 0.1, *p* = 0.797, Supplementary Table S5) and did not differ among sex ratios (*F*_2,73_ = 0.0, *p* = 0.958, Figure 3g, Supplementary Table S5).

### Sperm competition intensity and sexual networks

The sperm competition intensity index (Table 1) of all mated males (focal and rival) was greatest in male-biased groups (*n* males = 257, $\chi_{2}^{2}$ = 28.1, *p* < 0.001, Supplementary Table S6, Tukey’s HSD, female vs male-biased *p* < 0.001, equal vs male-biased *p* < 0.001), because females were typically more polyandrous in male-biased groups (Figures 4a–b, Supplementary Figure S2–S3 and S9). Focal males with higher sperm competition index values had lower reproductive success when controlling for their number of mates (*F*_1,49_ = 16.9, *p* < 0.001, Supplementary Table S7) and this relationship was consistent across sex ratios (*F*_2,45_ = 2.4, *p* = 0.106, Supplementary Table S7). Likewise, in all sex ratios, focal males facing a higher sperm competition index experienced decreased paternity share ($\chi_{1.49}^{2}$ = 36.1, *p* < 0.001) and this pattern did not vary across sex ratios ($\chi_{2.47}^{2}$ = 3.0, *p* = 0.220, Figure 4c, Supplementary Table S8). Finally, for all males (focal and rival) we identified a negative relationship between male number of mates and the sperm competition index (i.e., the Sperm Competition Intensity Correlation, SCIC, Table 1, *n* = 257, $\chi_{2}^{2}$ = 8.4, *p* = 0.004, Figure 4d, Supplementary Table S9), indicating that males that mated with more females also mated with less polyandrous females, on average. However, we found no influence of sex ratio on SCIC (*n* = 257, $\chi_{1.49}^{2}$ = 0.9, *p* = 0.639, Figure 4d, Supplementary Table S9).

## Discussion

A common assumption in evolutionary biology is that sexual selection will be accentuated in males as the sex ratio becomes more male-biased (Emlen & Oring, 1977; Klug et al., 2010; Kokko et al., 2012; Kvarnemo & Ahnesjo, 1996). We examined this assumption by assessing pre and postcopulatory sexual selection in experimental groups of *Drosophila melanogaster* held at different sex ratios. Our results indicate that while a male-biased sex ratio increased the opportunity for selection on males, there is no support for stronger Bateman gradients under a male-biased sex ratio. Overall, our results are consistent with recent theory that challenges the assumption that a male-biased sex ratio predictably drives stronger sexual selection on males. The opportunity for selection in males (*I*_*T*_) was greatest in male-biased populations. This increase was largely due to a greater standardized variance in male polygyny (i.e., the opportunity for precopulatory sexual selection on mate number, *I*_*M*_). These estimates indicate a greater potential for sexual selection to operate on males in male- vs. female-biased sex ratios, consistent with classical sexual selection theory. However, our null models also revealed that the tendency for an increased male *I*_*M*_ in male-biased groups is expected under random patterns of mating alone. These results support recent theory that demonstrates that stochastic variation in male mating alone can generate systematically higher opportunities for precopulatory selection at biased sex ratios (Jennions et al., 2012; Klug & Stone, 2021). This is particularly important given that this previous theory also shows that higher *I*_*M*_ values at male-biased sex ratios can occur even when the strength of sexual selection on competitive male mating traits does not change with sex ratio (Jennions et al., 2012). Our findings therefore suggest that there is no need to invoke mechanisms of increased competitive exclusion by males in male-biased groups to explain the higher opportunities for selection on males, which could instead be explained by a greater role of chance in mating in groups with lower average male mating frequencies. This finding is particularly relevant given experimental studies that manipulate adult sex ratios, rarely attempt to compare their results with null expectations (Jennions et al., 2012). This outcome underlines both the value of null models in comparing opportunities for selection across social contexts (Carleial et al., 2023; Jennions et al., 2012) and the need for research that estimates selection on phenotypes alongside measures of reproductive variance.

It is possible that the standardized rearing conditions of our experiment minimized variation in reproductive performance among males, and that more variable competitive rearing conditions would increase condition-dependent variation among males (Bath et al., 2023; Janicke et al., 2015). This might have increased the variation in male mating success observed in our study. However, despite standardized conditions we identified differences between focal and rival males in mating and reproductive success, and these patterns appeared inconsistent across sex ratios. These results suggest that focal males may be at a mating or reproductive disadvantage in female-biased or equal sex ratios, but that this disadvantage disappeared under the presumably more competitive male-biased situation, where both rival and focal males had equal success. These results further undermine the simplified expectation that male-biased sex ratios straightforwardly accentuate patterns of sexual selection on males and underline the need for future studies to measure selection on multiple male phenotypic traits to assess how sex ratio variation may favor and disfavor different traits in complex ways.

In addition to the opportunity for selection, we examined Bateman gradients. Counter to classical expectations (Janicke & Morrow, 2018), we found that male Bateman gradients did not vary across sex ratios, indicating that the force of selection on male mate acquisition did not vary from female- to male-biased sex ratios. Combined with our opportunity analyses, these results suggest that male-biased sex ratios did not increase the ability of some males to monopolize fertilizations. Our results provide an interesting parallel to a recent meta-analysis investigating the relationship between sex ratios and both $\beta_{M}$ and the opportunity for precopulatory selection on mate number (*I*_*M*_) (Janicke & Morrow, 2018). Using data from 58 species, this study indicated that the operational sex ratio positively predicted male *I*_*M*_ but was unrelated to male Bateman gradients. Our results provide complementary evidence that at least part of the correlation between the operational sex ratio and *I*_*M*_ may be driven by a greater role of chance in determining male mating patterns in biased sex ratios.

The Jones index combines information from both the Bateman gradient and the opportunity for precopulatory selection on mate number (*I*_*M*_). It represents the maximum potential sexual selection gradient on a precopulatory trait (Henshaw et al., 2016). Our results show that the Jones index was larger for males in male- vs. female-biased sex ratios. Given that the Jones index is the product of the Bateman gradient and $\sqrt{I_{M}}$ (Table 1), this result is primarily driven by the systematic bias towards higher male *I*_*M*_ in male-biased sex ratios. Therefore, despite representing the maximum potential sexual selection gradient on a precopulatory trait, differences in this maximum are likely driven by stochastic variance across sex ratios. We suggest that future studies must account for systematic biases in *I*_*M*_ when interpreting variation in the Jones index across sex ratios.

Despite no increase in the strength of precopulatory sexual selection in male-biased groups, as measured by Bateman gradients, our results indicated a potential increase in the importance of postcopulatory competition in male-biased groups. A male-biased sex ratio was associated with an increase in average female polyandry, which may allow females to exert greater choice via cryptic postmating mechanisms. Moreover, our multivariate selection gradient approach suggested that the potential strength of selection on male postcopulatory traits was strongest in male-biased groups, when we controlled for variation in mate acquisition and partner fecundity. In female *D. melanogaster*, mating typically renders females temporarily unreceptive to further mating (Manning, 1962). Thus, the increased female mating rates we observed in male-biased groups are likely driven by greater male harassment, suggesting that females reduce resistance when intense harassment renders resistance uneconomic or ineffective (Lauer et al., 1996; Rowe, 1992; Wigby & Chapman, 2004). As a result of increased polyandry, males in male-biased groups faced a higher potential sperm competition intensity, on average. Across all sex ratios, more polygynous males also mated with less polyandrous females on average (i.e., a negative SCIC), suggesting that polygynous males are more likely to experience a lower intensity of sperm competition. These patterns likely strengthened the correlation between male success in acquiring mates and reproductive success, and therefore male Bateman gradients (McDonald & Pizzari, 2018). Previous work suggests that negative relationships between male polygyny and female polyandry (SCIC) should be more frequent when mating is limited to small, isolated groups (McDonald, 2023). Our results support and advance this finding by indicating that such negative mating assortment in small sexual networks appears robust to sex ratio variation.

Finally, our results demonstrate a lower intensity of sexual selection in females vs. males, consistent with sexual selection theory (Lehtonen, 2022; Parker, 2014) and previous studies in *D. melanogaster* (Bjork & Pitnick, 2006; Davies et al., 2023). Although both sexes compete for reproductive opportunities in *D. melanogaster*, intra-female competition largely occurs over food and oviposition sites (Bath et al., 2018; Nilsen et al., 2004; but see Gaspar et al., 2022), which would not have been captured in our study. In contrast to males, the opportunity for precopulatory selection on mate number (*I*_*M*_) in females was consistently lower than null expectations. This pattern suggests that the number of mates per female was more evenly distributed than expected by chance, given the total number of copulations observed in each group. Such patterns are expected if females mate enough to obtain sperm and then limit re-mating to avoid costs from excess copulations (i.e., via female refractoriness). Alternatively, the pattern could be driven by selection on males to copulate with never-mated females, or by both limited female re-mating and male-driven processes. Disentangling the mechanisms underlying the low variation in the number of mates among females may provide insight into how female and male strategies influence sociosexual networks and the spread of sexually transmitted pathogens (Ashby & Gupta, 2013).

In summary, our study provides a comprehensive experimental examination of sexual selection across sex ratios. Our results support classic predictions for sex differences in sexual selection in a key model species. Consistent with classic theory, we show that the potential for sexual selection on males is highest in male-biased conditions. However, these differences may largely result from the increased influence of stochastic factors, rather than competitive mechanisms. Our work contributes to the ongoing debate over sex ratios and sexual selection by demonstrating that although male-biased sex ratios increase the potential for sexual selection to operate on males through increased variance in reproductive success, this potential for selection may not reflect changes in the strength of sexual selection on male mate acquisition. The results indicate that caution is warranted over the long-standing assumption that sex ratio bias is a good proxy for sexual selection.

## Supplementary material

Supplementary material is available online at *Evolution Letters*.

qraf001_suppl_Supplementary_Figures_S1-S8_Tables_S1-S9

## Data Availability

Data supporting results have been archived at Figshare doi: 10.6084/m9.figshare.28062542
