# Supplementary material for: A male-biased sex ratio increases the opportunity for precopulatory sexual selection but does not change the Bateman gradient
Source: Evol Lett. 2025 Feb 14;9(3):324–34. doi: 10.1093/evlett/qraf001 (PMC12137052; doi:10.1093/evlett/qraf001)
Supplement: qraf001_suppl_Supplementary_Figures_S1-S8_Tables_S1-S9 [file qraf001_suppl_supplementary_figures_s1-s8_tables_s1-s9.docx]

**Supporting information for “A male-biased sex ratio increases the Opportunity for sexual selection but does not change the Bateman gradient”**

Table of Contents

[Supplementary Methods 2](#_Toc185501487)

[The effect of sex ratio on mating and reproduction 2](#_Toc185501488)

[Daily patterns in the Opportunity for precopulatory selection on mate number (*I_M_)* 2](#_Toc185501489)

[Supplementary Results 3](#_Toc185501490)

[The effect of sex ratio on mating and reproduction 3](#_Toc185501491)

[Daily patterns in the Opportunity for precopulatory selection on mate number (*I_M_)* 4](#_Toc185501492)

[Supplementary references 5](#_Toc185501493)

[Supplementary figures 6](#_Toc185501494)

[Figure S1. Focal versus rival copulations and reproduction. 6](#_Toc185501495)

[Figure S2. Sexual networks. 7](#_Toc185501496)

[Figure S3. The influence of sex ratio on male and female mating behaviour. 8](#_Toc185501497)

[Figure S4. The influence of sex ratio on male and female reproduction. 9](#_Toc185501498)

[Figure S5. Daily patterns in the Opportunity for precopulatory selection on mate number (*I_M_*). 10](#_Toc185501499)

[Figure S6. Opportunity for postcopulatory sexual selection on males. 11](#_Toc185501500)

[Figure S7. Opportunities for selection excluding males that mated with sterile females 12](#_Toc185501501)

[Figure S8. Focal male Bateman gradients ($\boldsymbol{\beta M}$). 13](#_Toc185501502)

[Figure S9. Focal male sperm competition intensity (SCI). 14](#_Toc185501503)

[Supplementary tables 15](#_Toc185501504)

[Table S1. 15](#_Toc185501505)

[Table S2. 15](#_Toc185501506)

[Table S3. 16](#_Toc185501507)

[Table S4. 16](#_Toc185501508)

[Table S5. 17](#_Toc185501509)

[Table S6. 17](#_Toc185501510)

[Table S7. 17](#_Toc185501511)

[Table S8. 18](#_Toc185501512)

[Table S9. 18](#_Toc185501513)

# Supplementary Methods

## The effect of sex ratio on mating and reproduction

We first tested the influence of sex ratio on overall reproduction, including overall total number of copulations between all flies per vial and the total number of offspring per vial, using (generalised) linear models [(G)LMs)]. For total number of copulations we used a Poisson error distribution and for total number of offspring we used a quasipoisson error distribution. Models included sex ratio and experimental block as categorical variables.

We next used (G)LMs to test the influence of sex ratio on mating and reproduction for males and females. For all males (focal and rivals), we evaluated sex ratio differences in mean number of mates per male and mean number of copulations per male with Gaussian linear models. We tested for sex ratio differences in the probability that males obtained even a single mating across all males (focal and rival) using a binomial GLM. We then evaluated sex ratio differences in focal male reproductive success (*T*) (total number of daughters) using a GLM with a quasiposson error distribution and paternity share (the proportion of daughters produced by all of a focal male’s female partners that he sired, *P*) using a GLM with a quasibinomial error distribution. All models included sex ratio and experimental block as categorical variables.

For all females we evaluated sex ratio differences in the mean number of mates per female and mean number of copulations per female using Gaussian linear models. Both mean number of mates per female and mean number of copulations were log transformed. In addition, we evaluated sex ratio differences in average female reproductive success (*T*) per vial, where individual female reproductive success is calculated as all offspring (i.e. sons and daughters). Average female reproductive success was square root transformed and the model used a Gaussian error distribution. Finally, we analysed the effect of sex ratio on female reproductive success per mating (by dividing female reproductive success by her number of copulations) using a Gaussian linear model. All models included sex ratio and experimental block as categorical variables.

We generated p-values using F-tests and likelihood ratio tests as appropriate.

## Daily patterns in the Opportunity for precopulatory selection on mate number (*I_M_)*

Classical sexual selection theory predicts that both adult sex ratios and operational sex ratios may influence sex roles and patterns of sexual selection (Emlen & Oring 1977; Jennions & Fromhage 2017; Kappeler *et al.* 2022; Klug *et al.* 2010; Kvarnemo & Ahnesjo 1996; Schacht *et al.* 2017, 2022). While our study manipulates adult sex ratio, we expect our adult sex ratio treatments to reflect operational sex ratios most strongly at the beginning of trials. After females mate for the first time and become refractory, we expect operational sex ratios in all treatments to become more male-biased (or less female-biased) as females become unreceptive post-mating. We expect the ordinal relationship among sex ratio treatments to have been preserved throughout the experiment. To explore how the Opportunity for precopulatory selection on mate number (*I_M_*, Table 1) varies over the three days of behavioural observations we calculated *I_M_* for males cumulatively over the three days. We calculated *I_M_* across all males for each vial separately utilisng mating data from either day 1, days 1-2 and days 1-3. Calculating *I_M_* in this cumulative manner has been reported as the most appropriate way to explore how daily patterns of mating accrue to influence the overall standardised variance in mating success (Carleial et al., 2023). Note that the average *I_M_* values for days 1-3 calculated this way are equal to those values presented in Figure 2d.

To examine whether differences between sex ratios were consistent across days, we tested whether average vial *I_M_* varied over the course of the experiment using a linear mixed-effects model with male *I_M_* per vial as the response variable. Explanatory variables included sex ratio and experimental block as categorical variables and day as a 3-level categorical variable (i.e., day 1, days 1-2 and days 1-3). We also included the interaction between the day and sex ratio to examine if *I_M_* varied differently over successive days for each sex ratio. The identity of vials was included as a random effect to account for repeated measures of individual vials.

# Supplementary Results

## The effect of sex ratio on mating and reproduction

Sex ratio strongly influenced mating activity. As expected, the total number of copulations varied between sex ratios ($\chi_{2,77}^{2}$ = 23.7, *p* < 0.001, Fig. S3a) and were highest under a female-biased sex ratio and lowest in male-biased sex ratios (Tukey's HSD, female vs male-biased *p* < 0.001, equal vs male-biased *P* < 0.001). Total number of copulations was not influenced by experimental block ($\chi_{2,77}^{2}$ = 0.3, *p* = 0.879). As expected, the total number of offspring per vial varied between sex ratios ($\chi_{2,77}^{2}$ = 210.0, *p* < 0.001, Fig. S4a) and was higher under a female-biased and equal sex ratio compared with a male-biased sex ratio (Tukey's HSD, all *p* < 0.05). Total number of offspring varied between experimental block ($\chi_{2,77}^{2}$ = 279.9, *p* < 0.001).

Across all males (focal and rival), adult sex ratio influenced the mean number of mating partners and was highest in female-biased sex ratios (*F*_2,77_ =166.7, *p* < 0.001, Tukey's HSD, all *p* < 0.001, Fig. S3b). Male mean number of mating partners was not influenced by experimental block (*F*_2,77_ = 0.2, *p* = 0.807). The mean number of copulations per male was also significantly higher in female-biased sex ratios (*F*_2,77_ =129.3, *p* < 0.001; Tukey's HSD, all *p* < 0.001, Figs S3b). Male mean number of copulations was not influenced by experimental block (*F*_2,77_ = 0.3, *p* = 0.707). The probability that males (focal and rival) mated at least once was lower under male-biased sex ratios (probability [95% CI] Male-biased = 0.541 [0.465, 0.615], Equal = 0.833 [0.751, 0.892], Female-biased = 0.939 [0.861, 0.974], $\chi_{2,77}^{2}$ = 57.2, *p* < 0.001). The probability that males (focal and rival) mated at least once was not influenced by experimental block ($\chi_{2,77}^{2}$ = 0.6, *p* = 0.412).

Focal male reproductive success largely reflected mating patterns. Focal male reproductive success (*T*) was higher under a female-biased compared with a male-biased sex ratio ($\chi_{2,77}^{2}$ = 18.0, *p* < 0.001, Tukey's HSD, female vs male-biased *p* < 0.001, Fig. S4b). Focal male reproductive success (*T*) varied between experimental blocks ($\chi_{2,77}^{2}$ = 26.5, *p* < 0.001). Focal male paternity share (*P*) was not influenced by sex ratio when paternity share was calculated over all of a male’s partners ($\chi_{2,51}^{2}$ = 2.1, *p* = 0.354; Fig. S4c) or when paternity share was calculated over only polyandrous female partners ($\chi_{2,34}^{2}$ = 0.4, *p* = 0.807). In neither case was paternity share influenced by experimental block (all female partners; $\chi_{2,51}^{2}$ = 2.5, *p* = 0.287, polyandrous female partners; $\chi_{2,34}^{2}$ = 1.4, *p* = 0.489).

In females, sex ratio also influenced mating, but in the opposite direction from males. Females had a higher average number of mates in male-biased sex ratios (*F*_2,77_ =13.3, *p* < 0.001, Tukey's HSD, female vs male-biased *p* < 0.001, equal vs male-biased *p* < 0.001, Fig. S3d). The average number of mates per female was not influenced by experimental block (*F*_2,77_ = 0.0, *p* = 0.971). Similarly, females had a higher average number of copulations in male-biased sex ratios (*F*_2,77_ = 13.2, *p* < 0.001; Tukey's HSD, female vs male-biased *p* < 0.001, equal vs male-biased *P* < 0.005, Figs S3e) and variation in the average number of copulations was highest in male-biased sex ratios, ranging between 1 to 4 copulations per female (Figs S3e). The average number of copulations per female was not influenced by experimental block (*F*_2,77_ = 0.4, *p* = 0.689).

Average female reproductive success was not influenced by sex ratio (*F*_2,77_ = 1.0, *p* = 0.358; Fig. S4d) but varied between experimental block (*F*_2,77_ = 239.8, *p* < 0.001). However, female reproductive success per mating was lower in male-biased conditions, where mating rates where highest (F_2,77_ = 3.9, *p* = 0.024; Tukey's HSD female vs male-biased *p* < 0.05, Fig. S4e). Female reproductive success per mating also varied between experimental block (F_2,77_ = 53.0, *p* < 0.01).

## Daily patterns in the Opportunity for precopulatory selection on mate number (*I_M_)*

Our analysis of daily patterns in the Opportunity for precopulatory selection on mate number (*I_M_)* indicated that the differences in *I_M_* between sex ratios on day 1 and days 1-2 were broadly similar to those identified over all three days combined, indicating higher values of *I_M_* in male-biased sex ratios ($\chi_{2}^{2}$= 50.3, *p* < 0.001, Fig S5). Moreover, we identified a significant interaction between day order and sex ratio ($\chi_{4}^{2}$ = 27.77, *p* < 0.001), which was largely driven by a reduction in *I_M_* over cumulative days in a subset of vials from male-biased sex ratios (Fig S5).

These results demonstrate that patterns reported in the main text (i.e. a higher *I_M_* in male biased sex ratios) are also present on early days of the experiment, and that differences in *I_M_* between sex ratios are somewhat reduced over cumulative days of mating (Fig S5). Similar patterns have recently been documented in multiple species, and are likely driven by cumulative patterns of mating between males and females leading to a reduction in variance in polygyny, typical of mating patterns in small animal groups (Carleial *et al.* 2023).

# Supplementary references

Carleial, R., Pizzari, T., Richardson, D.S. & McDonald, G.C. (2023). Disentangling the causes of temporal variation in the opportunity for sexual selection. *Nat Commun*, 14, 1006.

Emlen, S. & Oring, L. (1977). Ecology, sexual selection and the evolution of mating systems. *Science*, 197, 215–223.

Jennions, M.D. & Fromhage, L. (2017). Not all sex ratios are equal: the Fisher condition, parental care and sexual selection. *Phil. Trans. R. Soc. B*, 372, 20160312.

Kappeler, P.M., Benhaiem, S., Fichtel, C., Fromhage, L., Höner, O.P., Jennions, M.D., *et al.* (2022). Sex roles and sex ratios in animals. *Biol Rev*, 98, 462–480.

Klug, H., Heuschele, J., Jennions, M.D. & Kokko, H. (2010). The mismeasurement of sexual selection. *J. Evol. Biol.*, 23, 447–462.

Kvarnemo, C. & Ahnesjo, I. (1996). The dynamics of operational sex ratios and competition for mates. *Trends Ecol Evol*, 11, 404–408.

Schacht, R., Beissinger, S.R., Wedekind, C., Jennions, M.D., Geffroy, B., Liker, A., *et al.* (2022). Adult sex ratios: causes of variation and implications for animal and human societies. *Commun Biol*, 5, 1–16.

Schacht, R., Kramer, K.L., Székely, T. & Kappeler, P.M. (2017). Adult sex ratios and reproductive strategies: a critical re-examination of sex differences in human and animal societies. *Philos. Trans. Royal Soc. B*, 372, 20160309.

# Supplementary figures

Figure S1. Focal versus rival copulations and reproduction. (a) Circles and error bars show the mean and 95% confidence intervals for the proportion of all copulations that involved focal males from generalised linear models. (b) Circles and error bars show the mean and 95% confidence intervals for the proportion of all offspring produced by focal males from generalised linear models. Horizontal lines show the expected proportion of copulations or offspring by focal males assuming equal success among all males. Expected proportions are: FB = 0.333 (1 focal, 2 rivals), EQ = 0.25 (1 focal, 3 rivals) and MB = 0.167 (1 focal, 5 rivals).

Figure S2. Sexual networks. Plots show sexual networks from all studied vials. Red nodes are focal males, black nodes are rival males and white nodes are females. Links between nodes indicate pairs that mated and the thickness of links reflects the number of copulations between pairs. Node size is scaled to the number of unique mates per individual. Networks within solid black circles are from female-biased (FB) groups, equal (EQ) sex ratio networks within dashed black circles and male-biased (MB) networks within red circles.

Figure S3. The influence of sex ratio on male and female mating behaviour. (a) The effect of sex ratio on the total number of copulations per vial over all individuals. (b) The average number of mates per male for each vial (focal and rivals). (c) The average number of copulations per male in each vial (focal and rivals). (d) The average number of mates per female in each vial. (e) The average number of copulations per female in each vial. White points indicate values for each vial. Pink points and bars indicate overall means and 95% confidence intervals. FB = female-biased groups, EQ = equal sex ratio groups and MB = male-biased groups.

Figure S4. The influence of sex ratio on male and female reproduction. (a) The effect of sex ratio on the total number of offspring produced per vial. Pink points and bars indicate means and 95% confidence interval across vials (b) The number of offspring per focal male for each vial and (c) the paternity share (*P*) per focal male in each vial (i.e. the proportion of a males partners’ offspring that he sires). White points indicate values for each focal male in each vial and pink points and bars indicate means and 95% confidence intervals. (d) The average number of offspring per female in each vial and (e) the average number of offspring per mating per female in each vial. White points indicate mean values across females for each vial. Pink points and bars indicate grand means and 95% confidence intervals. FB = female-biased groups, EQ = equal sex ratio groups and MB = male-biased groups.

Figure S5. Daily patterns in the Opportunity for precopulatory selection on mate number (*I_M_*). Plots show the values for the opportunity for precopulatory selection on mate number (*I_M_*, Table 1) for each sex ratio treatment calculated over cumulative days of the experiment (i.e. day 1 only, days 1-2 and days 1-3). Pink points and bars show mean values across all vials and 95% confidence intervals. Points connected by lines show the individual *I_M_* values for individual vials. FB = female-biased groups, EQ = equal sex ratio groups and MB = male-biased groups. Point shading indicates point overlap.

Figure S6. Opportunity for postcopulatory sexual selection on males. The opportunity for postcopulatory sexual selection (*I_P_*, Table 1) calculated as a focal male’s paternity share of the daughters produced across only polyandrous female mates (i.e., female mates that also mated with a rival male), for female-biased (FB), equal sex ratio (EQ), and male-biased groups (MB). Error bars show bootstrapped 95% confidence intervals.

Figure S7. Opportunities for selection excluding males that mated with sterile females**.** Panels show (a) the opportunity for selection (*I_T_*), (b) the opportunity for precopulatory selection on partner fecundity (*I_N_*) and (f) the opportunity for postcopulatory selection (*I_P_*) (see Table 1), for female-biased (FB), equal sex ratio (EQ), and male-biased groups (MB). Data exclude the reproductive success of two males from the equal sex ratio treatment that each monogamously mated with a unique female that produced a total of 0 and 1 offspring, respectively (i.e., two males that mated only with sterile females). In all cases results are qualitatively similar to those in the main text, although opportunity estimates under equal sex ratios tended to be lower and associated confidence intervals tended to be narrower. Error bars show bootstrapped 95% confidence intervals.

Figure S8. Focal male Bateman gradients ($\beta_{M}$). Plots show the mean-standardised Bateman gradients represented in a summarised from in figure 3a. Lines represent linear model predictions and shaded areas show 95% confidence intervals. FB = female-biased groups, EQ = equal sex ratio groups and MB = male-biased groups. Each point represents a value for a focal male.

Figure S9. Focal male sperm competition intensity (SCI). Violin plots show the sperm competition intensity (SCI) of focal males only. Pink points and bars show means and 95% confidence intervals. White points show values for each focal male.

# Supplementary tables

| Table S1. Results for the linear model assessing focal male Bateman gradients across sex ratio treatments. P-values are generated from F-tests. Experimental block is a 3-level categorical variable. Sex ratio is a 3-level categorical variable (i.e. Female-biased, Equal and Male-biased). Total vial productivity represents the total offspring produced per vial. Male reproductive success (*T*), mate number (*M*) and vial productivity are mean standardised. | | | | |
| --- | --- | --- | --- | --- |
| **Response** | **Variable** | **F-value** | ***d.f.*** | ***p*-value** |
| Reproductive success (*T*) | Mate number (*M*) | 70.996 | 1 | <0.001 |
|  | Sex ratio | 0.000 | 2 | 1 |
|  | Total vial productivity | 10.895 | 1 | 0.001 |
|  | Experimental block | 0.081 | 2 | 0.922 |
|  | Mate number (*M*) $\times$ Sex ratio | 0.223 | 2 | 0.801 |

| Table S2. Results for the linear model assessing focal male Jones Index across sex ratio treatments. P-values are generated from F-tests. Experimental block is a 3-level categorical variable. Sex ratio is a 3-level categorical variable (i.e. Female-biased, Equal and Male-biased). Total vial productivity represents the total offspring produced per vial. Male reproductive success (*T*) and vial productivity are mean standardised. Male mate number (*M*) is variance standardised. | | | | |
| --- | --- | --- | --- | --- |
| **Response** | **Variable** | **F-value** | ***d.f.*** | ***p*-value** |
| Reproductive success (*T*) | Mate number (*M*) | 61.461 | 1 | <0.001 |
|  | Sex ratio | 0.001 | 2 | 0.999 |
|  | Total vial productivity | 10.578 | 1 | 0.002 |
|  | Experimental block | 0.134 | 2 | 0.875 |
|  | Mate number (*M*) $\times$ Sex ratio | 2.789 | 2 | 0.068 |

| Table S3. Results for the linear model assessing variance-standardised multivariate selection gradients that measure the effect of one component (e.g., mate number, *M*) on male reproductive success (*T*) while controlling for the effect of the other components (e.g., partner fecundity *N* and paternity share *P*). P-values are generated from F-tests. Experimental block is a 3-level categorical variable. Sex ratio is a 3-level categorical variable (i.e. Female-biased, Equal and Male-biased). Male reproductive success (*T*) is mean-standardised. Male *M*, *N* and *P* are variance- standardised. We controlled for any differences in female fecundity between vials using residuals from the regression of *N* on total vial productivity, due to a strong correlation between vial productivity and the fecundity of female partners. | | | | |
| --- | --- | --- | --- | --- |
| **Response** | **Variable** | **F-value** | ***d.f.*** | ***p*-value** |
| Reproductive success (*T*) | Mate number (*M*) | 45.934 | 1 | <0.001 |
|  | Partner fecundity (*N*) | 33.293 | 1 | <0.001 |
|  | Paternity share (*P*) | 53.167 | 1 | <0.001 |
|  | Sex ratio | 0.031 | 2 | 0.969 |
|  | Residuals of vial productivity on *N* | 6.500 | 1 | 0.014 |
|  | Experimental block | 0.876 | 2 | 0.423 |
|  | Mate number (*M*)$\times$ Sex ratio | 0.095 | 2 | 0.909 |
|  | Partner fecundity (*N*) $\times$ Sex ratio | 0.259 | 2 | 0.773 |
|  | Paternity share (*P*) $\times$ Sex ratio | 3.143 | 2 | 0.054 |

| Table S4. Results for the linear model assessing focal female Bateman gradients across sex ratio treatments. P-values are generated from F-tests. Experimental block is a 3-level categorical variable. Sex ratio is a 3-level categorical variable (i.e. Female-biased, Equal and Male-biased). Total vial productivity represents the total offspring produced per vial. Female reproductive success (*T*), mate number (*M*) and vial productivity are mean standardised. | | | | |
| --- | --- | --- | --- | --- |
| **Response** | **Variable** | **F-value** | ***d.f.*** | ***p*-value** |
| Reproductive success (*T*) | Mate number (*M*) | 0.054 | 1 | 0.816 |
|  | Sex ratio | 0.015 | 2 | 0.985 |
|  | Total vial productivity | 22.615 | 1 | <0.001 |
|  | Experimental block | 2.901 | 2 | 0.061 |
|  | Mate number (*M*)$\times$ Sex ratio | 0.048 | 2 | 0.953 |

| Table S5. Results for the linear model assessing female Jones Index across sex ratio treatments. P-values are generated from F-tests. Experimental block is a 3-level categorical variable. Sex ratio is a 3-level categorical variable (i.e. Female-biased, Equal and Male-biased). Total vial productivity represents the total offspring produced per vial. Reproductive success (*T*) and vial productivity are mean standardised. Mate number (*M*) is variance standardised. | | | | |
| --- | --- | --- | --- | --- |
| **Response** | **Variable** | **F-value** | ***d.f.*** | ***p*-value** |
| Reproductive success (*T*) | Mate number (*M*) | 0.066 | 1 | 0.797 |
|  | Sex ratio | 0.015 | 2 | 0.985 |
|  | Total vial productivity | 22.506 | 1 | <0.001 |
|  | Experimental block | 2.907 | 2 | 0.061 |
|  | Mate number (*M*) $\times$ Sex ratio | 0.042 | 2 | 0.958 |

| Table S6. Results for the linear mixed-effects model assessing the effect of sex ratio treatments on male sperm competition intensity index (SCI). Male SCI was square root transformed. P-values are generated from likelihood ratio-tests. Experimental block is a 3-level categorical variable. Sex ratio is a 3-level categorical variable (i.e. Female-biased, Equal and Male-biased). Vial identity was included as a random effect. | | | | |
| --- | --- | --- | --- | --- |
| **Response** | **Variable** | $\boldsymbol{\chi}^{\boldsymbol{2}}$ | ***d.f.*** | ***p*-value** |
| Sperm competition intensity index (SCI) | Sex ratio | 28.057 | 2 | <0.001 |
|  | Experimental block | 0.392 | 2 | 0.822 |

| Table S7. Results for the linear model assessing the effect male competition intensity index (SCI) on male reproductive success (*T*) across sex ratios . P-values are generated from F-tests. Experimental block is a 3-level categorical variable. Sex ratio is a 3-level categorical variable (i.e. Female-biased, Equal and Male-biased). Total vial productivity represents the total offspring produced per vial. Male reproductive success (*T*), mate number (*M*), Sperm competition intensity index (SCI) and vial productivity are mean standardised. | | | | |
| --- | --- | --- | --- | --- |
| **Response** | **Variable** | **F-value** | ***d.f.*** | ***p*-value** |
| Reproductive success (*T*) | Sperm competition intensity index (SCI) | 16.902 | 1 | <0.001 |
|  | Mate Number (*M*) | 26.215 | 1 | <0.001 |
|  | Total vial productivity | 20.933 | 1 | <0.001 |
|  | Sex ratio | 0.020 | 2 | 0.980 |
|  | Experimental block | 0.645 | 2 | 0.529 |
|  | SCI $\times$ Sex ratio | 2.356 | 2 | 0.106 |
|  | Mate Number (*M*) $\times$ Sex ratio | 1.398 | 2 | 0.258 |

| Table S8. Results for the linear model assessing the effect male sperm competition intensity index (SCI) on male paternity share (*P*) across sex ratios. P-values are generated from likelihood ratio tests. Experimental block is a 3-level categorical variable. Sex ratio is a 3-level categorical variable (i.e. Female-biased, Equal and Male-biased). Total vial productivity represents the total offspring produced per vial. Mate number (*M*), Sperm competition intensity index (SCI) and vial productivity are mean standardised. | | | | |
| --- | --- | --- | --- | --- |
| **Response** | **Variable** | $\boldsymbol{\chi}^{\boldsymbol{2}}$ | ***d.f.*** | ***p*-value** |
| Paternity share (*P*) | Sperm competition intensity index (SCI) | 36.134 | 1 | <0.001 |
|  | Sex ratio | 0.295 | 2 | 0.863 |
|  | Total vial productivity | 1.533 | 1 | 0.0215 |
|  | Experimental block | 0.206 | 2 | 0.902 |
|  | SCI $\times$ Sex ratio | 3.028 | 2 | 0.220 |

| Table S9. Results for the linear mixed-effects model assessing the relationship between male mate number (*M*) on his sperm competition intensity index (SCI) across sex ratios. P-values are generated from likelihood ratio tests. Experimental block is a 3-level categorical variable. Sex ratio is a 3-level categorical variable (i.e. Female-biased, Equal and Male-biased). Total vial productivity represents the total offspring produced per vial. Mate number (*M*) and the Sperm competition intensity index (SCI) are mean standardised. Vial identity was included as a random effect. | | | | |
| --- | --- | --- | --- | --- |
| **Response** | **Variable** | $\boldsymbol{\chi}^{\boldsymbol{2}}$ | ***d.f.*** | ***p*-value** |
| Sperm competition intensity index (SCI) | Mate number (*M*) | 8.444 | 1 | 0.004 |
|  | Sex ratio | 0.322 | 2 | 0.852 |
|  | Experimental block | 0.613 | 2 | 0.736 |
|  | Mate number (*M*) $\times$ Sex ratio | 0.897 | 2 | 0. 639 |
